# Choice-making in an adaptive learning system with motivational pedagogical agents

**DOI:** 10.1038/s41539-025-00366-7

**Published:** 2025-11-18

**Authors:** Man Su, Belle Dang, Andy Nguyen, Tomohiro Nagashima

**Affiliations:** 1 Saarland University, Saarland Informatics Campus, Department of Computer Science, Saarbrücken, Germany; 2 University of Oulu, Learning & Educational Technology Research Unit (LET), Oulu, Finland

**Keywords:** Education, Education

## Abstract

Adaptive learning systems increasingly employ pedagogical agents (PAs) to enhance students’ engagement and learning outcomes, yet little is known about how motivational PAs influence students’ conceptual understanding and strategic choice-making. This study compared 49 school students (at 9th and 10th grade) using an adaptive algebra learning environment with either motivational PAs or instructional prompts (non-PAs). Results revealed that while all students demonstrated learning gains, prior knowledge significantly moderated outcomes. Lower-knowledge students achieved the greatest gains through reflective engagement with foundational tasks, whereas higher-knowledge students often adopted intuitive but error-prone strategies. Notably, process mining and lag sequential analysis revealed distinct choice-making trajectories, uncovering how motivational PAs influenced self-regulation patterns over time. This study advances the field by operationalizing choice-making as a measurable self-regulated learning construct and reframing strategic disengagement as an adaptive, agentic behavior. Findings underscore the importance of designing adaptive systems that support both content mastery and strategic choice-making.

## Introduction

A central goal of education is to cultivate self-regulated learners capable of making strategic and adaptive choices that enhance learning^1,2^. Choice-making, a critical self-regulated learning (SRL) component, reflects learners’ cognitive and metacognitive processes^3,4^. However, learners’ choices vary significantly based on performance levels and prior knowledge^5^.

High-performing students, those actively engaged and achieving strong learning gains, tend to select challenging tasks (e.g., quizzes, design reformulation) and strategically use scaffolds (e.g., evaluating visual aids) to optimize learning outcomes^6–8^. In contrast, low-performing students, those passively engaged or disengaged and achieving low learning gains, often avoid challenges, misuse scaffolds, and react impulsively to errors rather than engaging in metacognitive planning^9,10^.

Prior knowledge further shapes these behaviors. Learners with limited foundational knowledge struggle with complex tasks, yet targeted scaffolding and adaptive feedback can help foster high performance^11,12^. Conversely, high-prior-knowledge (HPK) learners may choose to disengage if tasks lack meaningful challenge^13^. This raises a critical question: How can educational systems scaffold low-prior-knowledge (LPK) learners to become high-performing students while supporting HPK learners to sustain their high performance through strategic choice-making?

Adaptive learning systems, such as Intelligent Tutoring Systems (ITS), offer promising solutions by embedding adaptive scaffolds that guide choice-making through real-time feedback, metacognitive prompts, and personalized learning pathways. For instance, Betty’s Brain provides strategic hints (viz., hints that support debugging and assessment of causal models) that encourage learners to monitor performance, reflect on actions, and make informed decisions^7^. Similarly, MetaTutor supports goal-setting and self-monitoring via metacognitive prompts and interactive dashboards^14,15^. Notably, many adaptive platforms operationalize these features through pedagogical agents (PAs) – interactive entities designed to simulate human-like guidance^16,17^. These agents enhance engagement by delivering motivational prompts, scaffolding complex tasks, and personalizing feedback.

Despite the integration of PAs into adaptive learning systems, a critical gap remains in understanding how PAs influence students’ choice-making behaviors, particularly in relation to their prior knowledge. While systems such as Betty’s Brain and MetaTutor provide motivational and instructional scaffolds, their impact on decision-making processes is often examined through aggregated metrics such as task completion rates rather than granular process data like problem-solving pathways, hint request frequency, and persistence after errors. This limitation prevents a nuanced understanding of how learners dynamically interact with PAs, highlighting the need for research that captures the fine-grained mechanisms underlying choice-making within adaptive learning environments.

To address this gap, this study investigates how PAs with motivational prompts influence conceptual understanding and strategic choice-making in an algebra-focused adaptive learning system. Specifically, we explore the following research questions:

RQ1: How do PAs with motivational prompts affect students’ conceptual understanding, moderated by prior knowledge?

RQ2: How do these PAs shape strategic choice-making across tasks, moderated by prior knowledge?

RQ3: How do prior knowledge levels dynamically influence choice-making processes over time?

Self-regulated learning (SRL) involves cognitive, metacognitive, motivational, and behavioral processes that learners use to plan, monitor, and regulate their learning^1,18^. Within Zimmerman’s cyclical SRL model, choice-making emerges as a process that bridges the performance and self-reflection phases. During performance, learners monitor their progress and evaluate their understanding; in self-reflection, they use this information to adapt strategies and decide on their next steps. Choice-making operationalizes this transition as it is the moment when learners act on their monitoring by deciding, for example, whether to attempt an optional task, change their problem-solving approach, or revisit a resource. In our study, this process is measured as the frequency of engagement with optional tasks in an adaptive learning system, providing an observable indicator of learners’ in-situ regulation and agency.

While grounded in Zimmerman’s model, choice-making can also be further interpreted through Oppezzo and Schwartz’s^19^ four-stage behavior change framework (“pre-intend,” “intend,” “implement,” and “inhabit” stages), which describes how strategic actions can evolve from initial consideration to stable, self-sustaining practices. In the context of our study, inhabiting a choice-making strategy does not imply automatic repetition without reflection; rather, it refers to the internalization of strategic reasoning at each choice opportunity where the decision is to engage with a task or to strategically disengage from it. This view aligns with SRL’s emphasis on adaptive expertise^2^, where learners can continue making effective, flexible decisions or strategic choices without ongoing support and adapt their strategic thinking to novel situations.

Adaptive learning systems, including intelligent tutoring systems (ITS), increasingly support SRL through real-time scaffolding and feedback^15,20^. Research in these systems has extensively explored *goal-setting*^21^, *self-monitoring*^11^, and *help-seeking*^10^, yet choice-making, an essential aspect of SRL, remains comparatively understudied. This gap arises partly from the tension between traditional adaptive system design principles and the complexity of modeling choice-making behaviors. Historically, systems such as the Cognitive Tutor and other ITSs have prioritized structured learning sequences that guide learners through predefined pathways to optimize skill mastery^22^. Although such approaches are effective for delivering adaptive feedback, error correction, and resource recommendations^13,23^, they often restrict learners’ autonomy in selecting tasks, strategies, or resources^21,24^. One notable exception is Roll et al.’s^10^ Help Tutor, which embedded metacognitive feedback on help-seeking into the Cognitive Tutor and demonstrated that learners can be supported to make more deliberate, self-regulated choices about whether, when, and how to seek assistance.

Additionally, choice-making itself poses significant modeling challenges. It is highly context-dependent, influenced by dynamic factors such as motivation, prior knowledge, and perceived task difficulty^25,26^. Unlike binary tasks with clear right or wrong answers, choice behaviors require nuanced assessments of learners’ adaptive capacities^4^ and flexibility in problem-solving^27^. This complexity has created both technical and theoretical hurdles for integrating choice-making into adaptive learning environments.

In the broader SRL landscape, choice-making shares similarities with other self-regulatory processes but is best understood as a broader construct that encompasses certain behaviors such as goal-setting, self-monitoring, help-seeking, and task selection. Similar to goal-setting, choice-making involves aligning potential actions with learning objectives; however, while goal-setting establishes the target^28^, choice-making focuses on the in-the-moment decisions about how to pursue, revise, or adapt smaller goals toward the end target^29^. It also builds on self-monitoring since learners must evaluate their progress to make informed decisions. Yet, self-monitoring is primarily evaluative, whereas choice-making requires translating that evaluation into action or a strategic choice not to act^30^. Help-seeking can be viewed as one specific form of choice-making, where the selected action is to obtain assistance from peers, instructors, or the system^31^. Similarly, task selection, often framed as choosing between tasks of varying difficulty^32^, is a particular manifestation of choice-making focused on selecting the next task. In contrast, the broader construct of choice-making extends beyond these specific behaviors to include persisting independently, switching strategies mid-task, or engaging with different resources within or across tasks.

A related and equally important construct is strategic disengagement, defined as a learner’s intentional decision not to implement a strategy or engage with an opportunity when doing so is unlikely to yield additional benefit^33^. For example, a student may decide not to request a scaffold when solving a familiar problem type because they are confident in their ability to succeed independently. Such disengagement differs from avoidance due to low motivation; it reflects metacognitive evaluation and selective effort investment, both of which are essential for developing adaptive expertise.

Neglecting choice-making in adaptive systems risks overlooking how learners develop adaptive expertise, which is the capacity to apply knowledge flexibly across novel contexts^2,4^. Strategic choice-making involves weighing whether to persist independently, seek help, revise work, or attempt alternative strategies^9,34^. However, many adaptive platforms still prioritize efficiency (e.g., rapid error correction) over autonomy, which can inadvertently encourage dependence on system guidance rather than fostering self-regulated learning^35,36^.

Recent evidence highlights the value of embedding structured opportunities for choice in adaptive systems. For example, Nagashima and colleagues ^8,37^ found that students in an algebra tutor who received metacognitive scaffolding chose to use visual supports more strategically, improving their algebra performance. Similarly, in Posterlet, learners who actively sought critical feedback and revised their work outperformed their peers^9,38^, demonstrating that choice-making can enhance engagement and learning outcomes^3^.

In adaptive learning systems, theoretically grounded prompts can encourage learners to engage in high-level SRL activities (e.g., self-explanation, solution comparison) and persist through challenges^20^. While prior systems like Betty’s Brain and MetaTutor have offered cognitive or metacognitive scaffolds^7,15^, few have explicitly emphasized the motivational value of strategic choice-making (e.g., “Choosing challenging tasks builds expertise”). Our study addresses this gap by embedding expectancy-value-informed prompts in pedagogical agents to scaffold both engagement and strategic disengagement, helping learners progress from momentary choices to sustained, self-regulated learning habits. In the following section, we detail the theoretical foundations for designing these motivational prompts, focusing on how Expectancy-Value Theory^39^ guided their development and application within our system.

In adaptive learning environments, prompts delivered by pedagogical agents (PAs) offer an opportunity to influence learners’ motivation and SRL behaviors (e.g., goal-setting, metacognitive reflection) in real time through targeted feedback and theory-driven messages^20,40,41^. Research has shown that prompts grounded in theories such as attribution theory^42^, social-cognitive theory^43^, goal orientation theory^44^, expectancy-value theory^45^, and self-determination theory^25^ can enhance learners’ self-efficacy, interest, engagement, motivation, problem-solving, and learning outcomes in STEM contexts^46–51^.

While all these theories offer promising directions, we decided against implementing most of them in the present study. Self-determination theory, for example, presupposes intrinsic motivation, which is subjective, and integrating it into an intervention consisting of stand-alone prompt messages is challenging^17^. Attribution theory links motivation to personal beliefs, requiring nuanced adaptation over time^52^. Social-cognitive theory interventions often involve rich narratives or modeling sequences, which exceed the scope of our intervention^47^. Goal orientation theory requires differentiating between mastery- and performance-oriented learners to adapt prompts effectively^14^. To avoid introducing additional parameters and complexity, we selected Expectancy-Value Theory (EVT^39^) as the sole theoretical foundation for prompt design in this study.

EVT posits that learners’ motivation is shaped by two key factors: their expectancy for success (beliefs about their ability to succeed) and the value they place on the task^39,53^. Task value encompasses intrinsic value (enjoyment or interest), utility value (usefulness for current or future goals), attainment value (personal importance of success), and perceived cost^45,54^. Drawing on the framework, each prompt in our study was explicitly designed to target one or more of these motivational constructs. For example, prompts emphasizing the practical benefits of manual calculation (e.g., “Manual calculations are required in many exams. Regular practice prepares you optimally for this.”) align with attainment value, while prompts highlighting strategy flexibility (e.g., “If you know and can compare several methods, you are more flexible and can choose the best method depending on the problem.”) address utility value and, indirectly, expectancy for success. A complete list of prompts used in the study, categorized by their corresponding EVT constructs, is provided in Supplementary Information (see Supplementary Table 1).

Moreover, motivational prompts from PAs differ from non-agent prompts in several important ways. PAs can enhance immediacy by delivering prompts contextually and in direct response to learner actions, thus reinforcing motivation at critical moments. They also provide personalization by adapting the tone or content of prompts to learners’ progress and increase social presence through human-like behaviors such as gaze, gesture, and conversational language^50^. These qualities, often referred to as the persona effect, have been shown to increase learner engagement and positive attitudes toward the learning task.

Compared to static or system-generated text prompts, PA-delivered prompts may activate motivational mechanisms more effectively by simulating a supportive social interaction. For example, immediacy allows the agent to connect task value to the learner’s just-completed step, while social presence can make the value message feel more personally relevant. Studies have found that animated PAs can moderate learning outcomes depending on factors like prior knowledge, suggesting that their impact is not solely visual but linked to how motivational content is framed and timed^55^.

In this study, embedding EVT-informed prompts into PA interactions aimed to leverage these mechanisms to support learners’ expectancy beliefs and perceived task value, with the goal of enhancing both engagement and performance in an algebra-focused adaptive learning environment.

## Results

### Conceptual understanding (RQ1)

Overall, all students showed an improvement from pretest to posttest, with an average increase of 4.22 points. Descriptive statistics (Table 1) revealed that students in the Agent condition exhibited a trend towards higher pre-post learning gains (M = 5.27, SD = 4.07) and lower error rates (M = 1.29, SD = 0.97) compared to those in the Non-Agent condition (learning gains: M = 3.22, SD = 4.45; error rates: M = 1.32, SD = 1.14). ANCOVA results revealed that the intervention condition (Non-Agent vs. Agent) did not have a significant effect on either posttest scores (*p* = 0.189) or error rates (*p* = 0.668) after controlling for pretest scores. However, pretest score was a significant predictor of both posttest performance (*p* < 0.001) and error rates (*p* = 0.030), indicating that students’ prior knowledge strongly influenced their conceptual understanding, regardless of the assigned condition.

**Table 1:** Means and standard deviations of variables related to conceptual understanding

	Non-agent			Agent		
	Overall (*n* = 25)	LPK (*n* = 10)	HPK (*n* = 15)	Overall (*n* = 24)	LPK (*n* = 14)	HPK (*n* = 10)
Pretest	8.12 (5.15)	3.08 (1.99)	11.48 (3.56)	6.63 (5.46)	2.80 (2.13)	11.98 (3.90)
Posttest	11.34 (4.53)	8.63 (5.16)	13.15 (3.05)	11.90 (4.16)	10.00 (3.99)	14.55 (2.80)
Learning Gains	3.22 (4.45)	5.55 (5.27)	1.67 (3.11)	5.27 (4.07)	7.20 (3.60)	2.58 (3.11)
Error Rate	1.32 (1.14)	1.46 (1.14)	1.22 (1.17)	1.29 (0.97)	1.66 (1.03)	0.77 (0.62)

*LPK* low prior knowledge students, *HPK* high prior knowledge students,values in parentheses represent standard deviations.

**Fig. 1:**
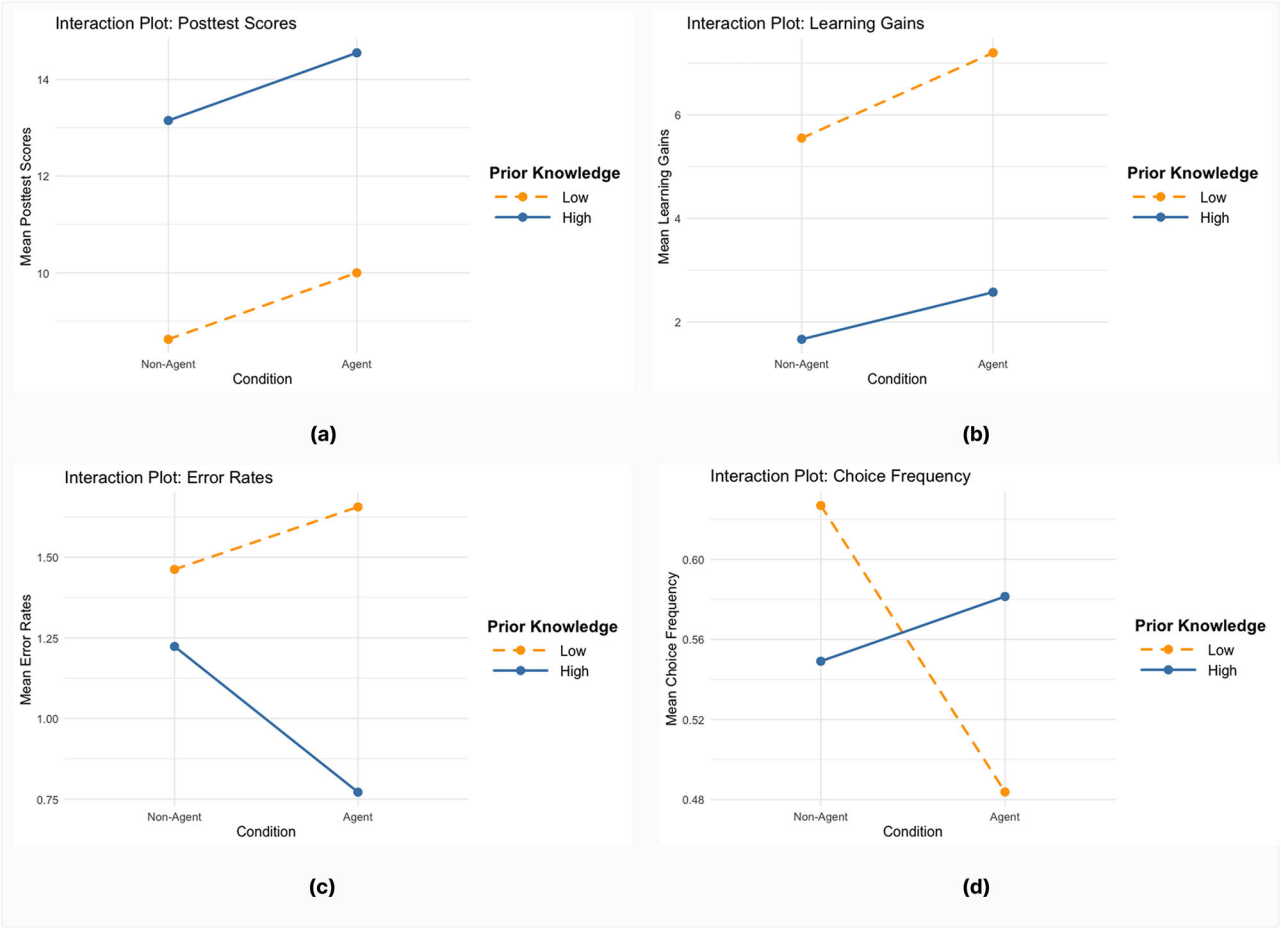
Interaction plots between prior knowledge and motivational agent scaffolding groups on posttest scores, learning gains, error rates, and choice frequency. This figure shows the interaction plots comparing students with high versus low prior knowledge across conditions with and without motivational agent scaffolding. Panel **a** illustrates posttest scores, where students with high prior knowledge in the agent condition scored higher than their peers in the non-agent condition, while low prior knowledge students showed more modest improvements. Panel **b** presents learning gains (posttest minus pretest scores), highlighting that low prior knowledge students exhibited larger gains in the agent condition compared to the non-agent condition, while high prior knowledge students demonstrated smaller relative gains. Panel **c** depicts error rates during the learning session, indicating that high prior knowledge students in the non-agent condition committed more errors, whereas students in the agent condition maintained lower error levels. Panel **d** displays choice frequency across tasks, showing that high prior knowledge students in the agent condition increased their task selection over time, while their peers in the non-agent condition decreased in frequency. Solid blue lines represent students with high prior knowledge, and dashed orange lines represent students with low prior knowledge.

To examine how the impact of pedagogical agents (PAs) on conceptual understanding varied by prior knowledge levels, we conducted two-way ANOVAs with condition (Agent vs. Non-Agent) and prior knowledge (HPK vs. LPK) as factors, analyzing posttest scores, learning gains, and error rates respectively. Results indicated a significant main effect of prior knowledge on posttest scores, F(1, 45) = 16.93, *p* < 0.001, showing that HPK students achieved significantly higher posttest scores compared to LPK peers. However, there was no significant main effect of condition F(1, 45) = 0.26, *p* = 0.611, nor was there a significant interaction between condition and prior knowledge F(1, 45) = 0.00, *p* = 0.991. Post-hoc pairwise comparisons (Tukey-adjusted) revealed that the Non-Agent LPK group scored significantly lower than both the Non-Agent HPK group (*p* = 0.027) and the Agent HPK group (*p* = 0.006). As depicted in Fig. 1a, HPK students consistently scored higher, though the Agent condition did not significantly outperform the Non-Agent condition.

For learning gains, there was also a significant main effect of prior knowledge, F(1, 45) = 14.98, *p* < 0.001, with LPK students demonstrating greater improvements due to lower baseline scores (see Table 1). The main effect of condition approached significance (F(1, 45) = 3.62, *p* = 0.064), indicating that students in the Agent condition showed a trend towards higher gains. However, there was no significant interaction effect (F(1, 45) = 0.11, *p* = 0.738). Post-hoc analyses indicated that the Agent LPK group achieved significantly higher learning gains compared to the Non-Agent HPK group (*p* = 0.002). Figure 1b illustrates that learning gains were greater for LPK students, particularly those in the Agent condition.

Regarding error rates, results showed a marginally significant main effect of prior knowledge, F(1, 45) = 3.45, *p* = 0.070, suggesting a trend where HPK students had lower error rates. There were no significant main effects of condition (F(1, 45) = 0.01, *p* = 0.916) or interaction effects (F(1, 45) = 1.16, *p* = 0.288). Post-hoc comparisons revealed no statistically significant differences between groups. However, Fig. 1c shows descriptively lower error rates among HPK students, especially in the Agent condition, suggesting a potential reduction of errors in this subgroup.

### Choice-making behaviors (RQ2)

On average, students engaged with optional tasks in 55% of the opportunities presented across all scenarios. In this context, “engagement” is defined as the proportion of available optional tasks a student chose to complete, out of the total number of such opportunities encountered. Thus, a value of 0.70 for S1_WorkEx means that, on average, students opted to engage with 70% of the worked-example opportunities they were given.

Although Fig. 1d suggests a possible interaction, two-way ANOVA showed no significant main effect of condition, F(1, 45) = 0.39, *p* = 0.537, or prior knowledge, F(1, 45) = 0.01, *p* = 0.924 on their choice frequency, and no significant interaction effect between condition and prior knowledge, F(1, 45) = 0.93, *p* = 0.341. Post-hoc pairwise comparisons also showed no statistically significant differences between groups. As visualized in Fig. 1d, choice frequency was higher for LPK students in the Non-Agent condition, but this pattern reversed in the Agent condition, where HPK students showed higher choice frequency. This pattern suggests that the effectiveness of motivational pedagogical agents may depend on students’ prior knowledge levels, which calls for a more detailed comparison of students’ choice-making behaviors across different choice-making scenarios.

A closer look at Table 2 reveals distinct engagement patterns. LPK students tended to prioritize foundational tasks: for worked examples (S1), engagement rates were higher in both Agent (M_LPK_ = 0.71) and Non-Agent (M_LPK_ = 0.70) conditions compared to HPK peers (M_HPK_ = 0.40–0.47). In contrast, HPK students engaged more often with advanced tasks regardless of conditions. For instance, HPK students showed higher engagement with applying the advanced equalization strategy (S7: M_HPK_ = 0.50–0.67) compared to LPK peers (S7: M_LPK_ = 0.33). Notably, Non-Agent LPK students exhibited unexpectedly high engagement in comparison tasks (S3, M_LPK_ = 0.74), possibly reflecting intrinsic motivation to explore alternatives without PA encouragement. Overall, HPK students in the Agent Condition engaged more frequently across all optional tasks (M_HPK_ = 0.58 vs. M_LPK_ = 0.48), whereas the opposite trend appeared in the Non-Agent Condition (M_HPK_ = 0.55 vs. M_LPK_ = 0.63, Fig. 1d).

**Table 2:** Descriptive statistics of students’ choice-making frequency across scenarios

	Non-agent			Agent		
	Overall (*n* = 25)	Low (*n* = 10)	High (*n* = 15)	Overall (*n* = 24)	Low (*n* = 14)	High (*n* = 10)
Overall	0.58 (0.29)	0.63 (0.28)	0.55 (0.31)	0.52 (0.33)	0.48 (0.33)	0.58 (0.34)
S1_WorkEx	0.56 (0.51)	0.70 (0.48)	0.47 (0.52)	0.58 (0.50)	0.71 (0.47)	0.40 (0.52)
S2_Self-Expl	0.52 (0.40)	0.58 (0.35)	0.49 (0.44)	0.54 (0.43)	0.55 (0.42)	0.53 (0.46)
S3_Comp	0.55 (0.43)	0.74 (0.37)	0.42 (0.43)	0.44 (0.37)	0.39 (0.35)	0.50 (0.39)
S4_Resolve	0.27 (0.47)	0.50 (0.58)	0.14 (0.38)	0.40 (0.49)	0.51 (0.50)	0.00 (0.00)
S5_1^st^ Sol	0.56 (0.31)	0.54 (0.30)	0.57 (0.33)	0.53 (0.38)	0.46 (0.37)	0.62 (0.40)
S6_2^nd^ Sol	0.64 (0.36)	0.66 (0.36)	0.63 (0.37)	0.53 (0.39)	0.47 (0.39)	0.60 (0.40)
S7_Adv_Equ	0.58 (0.52)	0.33 (0.58)	0.67 (0.50)	0.42 (0.52)	0.33 (0.52)	0.50 (0.55)
S8_Adv_Sub	0.20 (0.42)	0.00 (0.00)	0.25 (0.46)	0.27 (0.47)	0.17 (0.41)	0.40 (0.55)

Means and standard deviations of students’ choice-making frequency across different scenarios. Values represent the proportion of optional task opportunities within each scenario type that students chose to complete, out of the total opportunities encountered. Values in parentheses represent standard deviations.

*WorkEx* worked examples, *Self-Expl* self-explanations, *Comp* comparisons, *Resolve* resolving, *1st & 2nd Sol* 1st solution and 2nd solution, *Adv_Equ* advanced equalization, *Adv_Sub* advanced substitution.

### Dynamic choice-making processes (RQ3)

First, LSA was conducted to examine the micro-level, sequential dependencies in students’ choice-making behaviors to identify how HPK and LPK students differed in their decision-making tendencies during their learning tasks. Tables 3 and 4 display the transition probabilities matrix across various categories for students with high and low pretest score, respectively. A chi-square test verified a significant relationship between the rows and columns of the recorded frequencies for both high (χ² = 1967.63, df = 49, *p* < 0.001, Monte Carlo 2-sided) and low group (χ² = 1311.42, df = 49, *p* < 0.001, Monte Carlo 2-sided).

**Table 3:** Transition probabilities (high prior knowledge group)

Decision action	NoToExtra	AttemptSuccess	NoToPrompt	YesToPrompt	CorrectDecision	FailedAttempt	YesToExtra	IncorrectDecision
NoToExtra	0.00	0.86	0.00	0.00	0.00	0.14	0.00	0.00
AttemptSuccess	0.00	0.33	0.33	0.29	0.00	0.04	0.01	0.00
NoToPrompt	0.03	0.77	0.09	0.02	0.00	0.06	0.02	0.00
YesToPrompt	0.01	0.68	0.00	0.00	0.09	0.19	0.01	0.02
CorrectDecision	0.03	0.82	0.00	0.00	0.00	0.11	0.05	0.00
FailedAttempt	0.00	0.55	0.00	0.00	0.00	0.45	0.00	0.00
YesToExtra	0.00	0.38	0.13	0.42	0.00	0.08	0.00	0.00
IncorrectDecision	0.00	0.63	0.00	0.00	0.00	0.38	0.00	0.00

**Table 4:** Transition probabilities (low prior knowledge group)

Decision action	YesToExtra	AttemptSuccess	NoToPrompt	YesToPrompt	CorrectDecision	FailedAttempt	NoToExtra	IncorrectDecision
YesToExtra	0.00	0.71	0.19	0.00	0.00	0.10	0.00	0.00
AttemptSuccess	0.01	0.30	0.37	0.25	0.00	0.06	0.00	0.00
NoToPrompt	0.03	0.69	0.09	0.05	0.00	0.11	0.04	0.00
YesToPrompt	0.01	0.52	0.00	0.00	0.10	0.33	0.01	0.04
CorrectDecision	0.00	0.70	0.00	0.00	0.00	0.27	0.03	0.00
FailedAttempt	0.00	0.51	0.00	0.00	0.00	0.49	0.00	0.00
NoToExtra	0.00	0.65	0.00	0.00	0.00	0.30	0.05	0.00
IncorrectDecision	0.00	0.50	0.00	0.00	0.00	0.50	0.00	0.00

Based on the computation of z-values and Yule’s Q, we found the following sequences of two elements (lag 1) as statistically significant (that is z > 1.96 and Q > 0.30, see Table 5):

**Table 5:** Significant two element (lag 1) transitions by group

#	High prior knowledge group		Low prior knowledge group	
1	NoToPrompt *→* AttemptSuccess	(z = 13.20, Q = 0.62)	NoToPrompt *→* AttemptSuccess	(z = 10.20, Q = 0.54)
2	CorrectDecision *→* AttemptSuccess	(z = 3.83, Q = 0.63)	CorrectDecision *→* AttemptSuccess	(z = 2.70, Q = 0.48)
3	AttemptSuccess *→* YesToPrompt	(z = 19.20, Q = 0.92)	AttemptSuccess *→* YesToPrompt	(z = 16.18, Q = 0.90)
4	AttemptSuccess *→* NoToPrompt	(z = 19.62, Q = 0.86)	AttemptSuccess *→* NoToPrompt	(z = 19.56, Q = 0.89)
5	IncorrectDecision *→* FailedAttempt	(z = 2.30, Q = 0.64)	IncorrectDecision *→* FailedAttempt	(z = 2.93, Q = 0.62)
6	FailedAttempt *→* FailedAttempt	(z = 19.18, Q = 0.82)	FailedAttempt *→* FailedAttempt	(z = 16.58, Q = 0.74)
7	YesToPrompt *→* FailedAttempt	(z = 5.15, Q = 0.35)	YesToPrompt *→* FailedAttempt	(z = 5.85, Q = 0.39)
8	YesToPrompt *→* CorrectDecision	(z = 14.17, Q = 0.99)	YesToPrompt *→* CorrectDecision	(z = 2.38, Q = 0.50)
9	YesToPrompt *→* IncorrectDecision	(z = 6.67, Q = 1)	YesToPrompt *→* IncorrectDecision	(z = 5.85, Q = 0.39)
10	NoToPrompt *→* NoToExtra	(z = 5.56, Q = 0.75)	NoToPrompt *→* NoToExtra	(z = 5.94, Q = 0.83)
11	NoToPrompt *→* YesToExtra	(z = 2.38, Q = 045)	NoToPrompt *→* YesToExtra	(z = 3.44, Q = 0.63)
12	**NoToExtra** ***→*** **AttemptSuccess**	**(z** **=** **3.84, Q** **=** **0.72)**	*–*	*–*
13	**CorrectDecision** ***→*** **YesToExtra**	**(z** **=** **2.85, Q** **=** **0.45)**	*–*	*–*
14	**YesToExtra** ***→*** **YesToPrompt**	**(z** **=** **3.61, Q** **=** **0.60)**	*–*	*–*
15	**YesToPrompt** ***→*** **AttemptSuccess**	**(z** **=** **7.41, Q** **=** **0.39)**	*–*	*–*
16	–	*–*	**YesToExtra** ***→*** **AttemptSuccess**	**(z** **=** **2.38, Q** **=** **0.50)**

Boldface sequences represent patterns unique to each group.

Overall, both groups exhibited several common transitions, anticipated sequences within our learning task, reflecting the structured design of the exercises to guide students through core tasks while fostering reflective and strategic engagement. Notable shared transitions include #1 (NoToPrompt → AttemptSuccess) and #2 (CorrectDecision → AttemptSuccess), which highlights the capacity of students to complete tasks either independently or after making correct decisions. Similarly, sequences #3 (AttemptSuccess → YesToPrompt) and #4 (AttemptSuccess → NoToPrompt) illustrates students’ alternation between engaging with reflective prompts provided after successful attempts. These are designed to encourage deeper reflection and rationalization of correct decisions. For example, the HPK group seems to show higher frequency (z = 19.20, Q = 0.92) of engaging with optional tasks (i.e., self-explanation) than the LPK group (z = 16.18, Q = 0.90) after successfully selecting the most efficient method. Both groups also exhibited common patterns in error handling, such as sequences #5 (IncorrectDecision → FailedAttempt) and #6 (FailedAttempt → FailedAttempt). These behaviors reflect response patterns where errors are followed by rapid subsequent selections potentially before any substantial re-evaluation.

However, subsequent pathways diverged significantly between the groups, reflecting differing approaches to task management and progression by learners with different prior knowledge. The HPK group exhibited additional pathways not observed in the LPK group, reflecting their confidence-driven strategies and willingness to dynamically engage with tasks and prompts. The sequence #12 (NoToExtra → AttemptSuccess) demonstrated their ability to bypass preparatory tasks and proceed directly to task success. Similarly, sequence #13 (CorrectDecision → YesToExtra) and higher frequency of sequence #3 (AttemptSuccess → YesToPrompt, z = 19.20, Q = 0.92) highlighted that students in the HPK group, when achieving success, were more likely to engage in additional advanced exercises or voluntary tasks. This sequence suggests that, for this group, successful outcomes increased their willingness to take on further challenges, creating opportunities for deeper engagement. Additionally, sequence #15 (YesToPrompt → AttemptSuccess) demonstrated their successful application of insights gained from prompts, leading to task successful completion.

In contrast, the LPK group demonstrated a distinct preparatory approach, with the sequence #16 (YesToExtra → AttemptSuccess) being unique to this group. This pathway underscores their reliance on preparatory tasks (i.e., worked examples, which were presented at the beginning of the learning session) to build confidence and achieve success in core tasks.

We also conducted a LSA for a significant sequence of three elements. For the LPK group, the following chains were significant.

AttemptSuccess → YesToPrompt → CorrectDecision(z_lag2_ = 3.81, Q_lag2_ = 0.66)

YesToPrompt → CorrectDecision → AttemptSuccess(z_lag2_ = 7.83, Q_lag2_ = 0.49)

CorrectDecision → AttemptSuccess → YesToPrompt (z_lag2_ = 5.70, Q_lag2_ = 0.73)

NoToPrompt → AttemptSuccess → NoToPrompt(z_lag2_ = 10.78, Q_lag2_ = 0.57)

For the HPK group, the following chains were significant.

AttemptSuccess → YesToPrompt → CorrectDecision(z_lag2_ = 4.81, Q_lag2_ = 0.79)

CorrectDecision → YesToExtra → YesToPrompt(z_lag2_ = 10.53, Q_lag2_ = 0.90)

FailedAttempt → FailedAttempt → FailedAttempt(z_lag2_ = 4.33, Q_lag2_ = 0.33)

YesToPrompt → CorrectDecision → YesToExtra(z_lag2_ = 3.61, Q_lag2_ = 0.60)

YesToExtra → YesToPrompt → AttemptSuccess(z_lag2_ = 3.62, Q_lag2_ = 0.75)

NoToPrompt → AttemptSuccess → NoToPrompt (z_lag2_ = 14.01, Q_lag2_ = 0.63)

Both groups shared sequences like AttemptSuccess → YesToPrompt → CorrectDecision, highlighting their willingness to engage with prompts, such as those involving multiple-choice self-explanation tasks that facilitate deep reflection upon the choices made. In contrast, the shared sequence NoToPrompt → AttemptSuccess → NoToPrompt reflects their tendencies to bypass prompts, likely those requiring extended effort such as manual calculation.

For the LPK group, three-event sequences YesToPrompt → CorrectDecision → AttemptSuccess and CorrectDecision → AttemptSuccess → YesToPrompt indicate an increased willingness to engage with prompts after observing that such engagement often leads to successful outcomes. For the HPK group, sequences such as CorrectDecision → YesToExtra → YesToPrompt, and YesToPrompt → CorrectDecision → YesToExtra exhibit a success-driven engagement pattern. Specifically, success often led to increased willingness to take on extra advanced tasks. This pattern is consistent with their higher prior knowledge and confidence. However, HPK group’s sequence (FailedAttempt → FailedAttempt → FailedAttempt) suggests that some successes may have resulted from quick judgments of repeated failed attempts rather than thorough reflections or deep reasonings. Given the higher prior knowledge and confidence in this group, these learners may have been more inclined to rely on domain familiarity and heuristic reasoning, making them less inclined to pause for strategic reconsideration or seek additional support. This finding resonates with Roll et al.’s^10^ observations on the mechanism of unreflective repetition and help avoidance, with learners bypassing opportunities for metacognitive regulation, where learners persist in acting without revising their approach. This persistent error pattern was not observed in the LPK group, potentially indicating a more cautious and deliberative approach in the LPK learners’ decision-making processes.

**Fig. 2:**
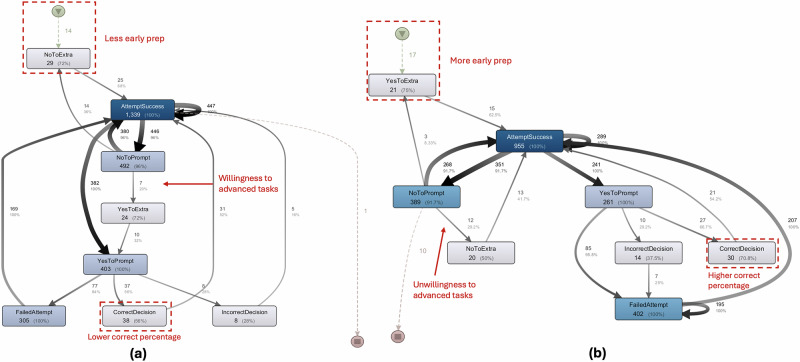
Process maps of decision-making patterns by prior knowledge group. This figure presents process mining maps generated with the Disco tool using 100% of activities and 0% of paths, which results in displaying only the most important connections. Panel (**a**) shows the high prior knowledge group, while panel (**b**) shows the low prior knowledge group. Nodes represent types of learning activities, and arrows represent transitions between activities, with arrow thickness scaled to transition frequency. Dashed arrows indicate links to actions occurring at the very beginning or end of the process. In panel (**a**), the high prior knowledge group demonstrates less preparatory activity at the start and greater willingness to transition directly into advanced tasks. In panel (**b**), the low prior knowledge group engages in more early preparation and shows a stronger tendency to avoid advanced tasks, reflecting more cautious decision-making. Additionally, the maps highlight that the low prior knowledge group achieved a higher percentage of correct decisions overall compared to their high prior knowledge peers. The shading of the nodes encodes activity frequency: dark blue boxes represent activities that occurred with higher frequency, while lighter blue boxes represent activities that occurred with lower frequency. Circles with green shading mark the start of the process, circles with red shading mark the end of the process, solid black arrows indicate process flows, and dashed arrows highlight activities connected to the very beginning or end of the session.

While LSA identifies critical micro-level patterns, process mining provides an overall view of how students navigated tasks and made choices during their learning sessions. This analysis revealed dominant pathways and differences between HPK and LPK students in their sequential choice-making behaviors (see Fig. 2). This analysis highlights absolute frequencies and case coverages to reveal the overarching tendencies in their interaction patterns within the learning environment.

Overall, the process mining analysis reveals that the HPK and LPK groups exhibit similar overarching patterns and comparable structures of transitioning between task attempts, prompts, and decision choices. This similarity aligns with the anticipated outcomes of our task design. Both groups share core loops, such as AttemptSuccess → NoToPrompt → AttemptSuccess, further supporting LSA’s findings of a common tendency to bypass certain prompts such as those requiring manual calculation efforts.

However, key differences emerge in how these two groups regulate their actions, handle failures, engage with preparatory tasks, and make decisions. The process map for the HPK group reveals a pattern of initially avoiding preparatory tasks (f_Start → NoToExtra_ = 72%), favoring direct progression to tasks with high success levels (AttemptSuccess, f_High_ = 1339). As their interactions unfolded, they increasingly engaged with more advanced tasks and prompts, such as in the pathway YesToExtra → YesToPrompt (f_High_ = 32%), demonstrating a willingness to tackle more complex activities after initial successes. At first glance, the process map suggests that failures in the HPK group frequently transitioned to AttemptSuccess, potentially indicating quick recovery from errors. However, this transition (Failed Attempt → AttemptSuccess) occurred less frequently in the HPK group (f_High_ = 169) compared to the LPK group (f_Low_ = 207). Furthermore, the significant three-event sequence FailedAttempt → FailedAttempt → FailedAttempt (z_lag2_ = 4.33, Q_lag2_ = 0.33) suggests that the dominant pathway observed in the process maps may be more reflective of the group’s higher task frequency and overall engagement with tasks rather than superior error regulation. This pattern highlights a defining characteristic of the HPK group: confidence-driven task engagement and a strong propensity for success. Their approach is often facilitated by intuitive, heuristic decision-making and knowledge familiarity, rather than systematic regulation strategies involving deeper error reflection.

In contrast, the LPK group engaged more frequently with preparatory tasks early on, as reflected in pathways like Start → YesToExtra (f = 75%), suggesting a focus on foundational activities to build confidence. However, as interactions progressed, NoToExtra pathways became increasingly prevalent (f_NoToPrompt → NoToExtra_ = 29.2%), reflecting a reduced willingness to engage with advanced tasks later. This contrasts with the HPK group, which increasingly engaged with advanced tasks midway, as seen in YesToExtra → YesToPrompt (f_High_ = 32%). Interestingly, the LPK group demonstrated a higher proportion of CorrectDecision outcomes compared to the HPK group (f_Low_ = 70.8%; f_High_ = 56%). This suggests a more cautious and reflective decision-making approach. While the LPK group exhibited a higher overall number of failures (f_Low_ = 402; f_High_ = 305), these behaviors align with expectations for a group with LPK. Their deliberate engagement with prompts, however, may have contributed to their more favorable ratio of correct decision outcomes.

## Discussion

This study explores how motivational pedagogical agents (PAs) influence students’ conceptual understanding and choice-making behaviors in adaptive learning systems, specifically among students with different levels of prior knowledge. Our findings contribute both theoretical insights and practical suggestions for designing adaptive learning systems that can better support diverse learners’ needs. Below, we reflect on key findings, theoretical and design implications, as well as limitations and future directions.

The study examined whether pedagogical agents (PAs) delivering motivational prompts could enhance students’ conceptual understanding and strategic decision-making. While descriptive patterns suggested that students in the Agent condition had slightly higher learning gains and lower error rates, these differences were not statistically significant after accounting for prior knowledge.

Several factors may help explain these null findings. First, the intervention duration was relatively short (25 min of system use), which may have limited the accumulation of measurable benefits from motivational prompts. Prior studies have shown that the impact of motivational scaffolding often emerges over longer or repeated exposures^17,50^. Second, the motivational prompts were not personalized beyond the choice-making scenario type. More adaptive tailoring, such as adjusting language complexity, providing targeted hints, or varying prompt frequency based on student responses, might have yielded stronger effects, particularly for LPK learners. Third, the prompts were delivered at fixed points within tasks, without dynamically adapting to indicators of disengagement or misunderstanding. Literature suggests that timing prompts in response to students’ in-task behaviors can enhance their motivational impact^7,56^. Finally, because both conditions provided the same cognitive and metacognitive choice-making opportunities, the added benefit of motivational framing alone may have been insufficient to produce statistically significant gains in a short-term study.

Despite the lack of significant main effects of condition, prior knowledge clearly shaped students’ learning outcomes. LPK students exhibited greater learning gains compared to the HPK counterparts (there does not appear to be a strong ceiling effect, as some HPK students still showed gains). This result aligns with existing research suggesting that learners with less initial knowledge often benefit more from scaffolding opportunities^5,21^. These findings suggest that combining motivational prompts with more targeted adaptive support may yield stronger benefits for LPK learners in future iterations.

Interestingly, although overall choice-making frequencies did not significantly differ between conditions, we observed nuanced differences in specific choice-making scenarios. Students with lower prior knowledge more often chose foundational tasks, such as worked examples, possibly indicating a need for building initial confidence before engaging in more complex tasks. Meanwhile, students with high prior knowledge readily engaged with more advanced, optional tasks when prompted. This reflects the nuanced role prior knowledge plays in shaping engagement patterns while students interact with the system^7^.

A major strength of this study is the detailed analysis of students' interactions using lag sequential analysis (LSA) and process mining, which allowed for a detailed examination of the micro-level and macro-level patterns in students’ choice-making behaviors. These methods offered a deeper understanding of how learners move through decision-making phases and how these transitions relate to their learning outcomes. For example, HPK students exhibited confidence-driven behaviors, bypassing preparatory tasks, and quickly jumping into challenging tasks after successes, though sometimes at the cost of repeated failures. In contrast, LPK students took a cautious and deliberate approach, which relied on foundational tasks such as going through worked examples to build confidence over time. This fine-grained analysis provides valuable insights into the temporal dynamics of SRL and reveals the diverse pathways that learners take as they engage with learning tasks dynamically within adaptive systems^57^.

Our study offers two significant theoretical contributions to the understanding of self-regulated learning (SRL) by providing new insights into the temporal dynamics of choice-making and the role of strategic disengagement in adaptive learning environments.

First, we examine choice-making not as a comprehensive operationalization of SRL but as a specific, measurable facet of SRL behavior that can be tracked and analyzed over time. By leveraging process mining methods to capture students’ engagement with optional tasks, we illustrate how choice-making unfolds as a context-sensitive, temporal-dynamic process, which is consistent with Zimmerman and Campillo^1^’s cyclical model of self-regulation and Oppezzo and Schwartz^19^’s staged behavior-change model. Rather than claiming to measure all forms of SRL choice (e.g., goal-setting, help-seeking), our analysis focuses on task-level engagement decisions within clearly defined scenarios. This finer-grained, temporal lens responds to calls by Azevedo et al.^15^ for methods that can capture SRL processes in situ and in sequence. Our findings further suggest that prior knowledge plays an important role in shaping these temporal choice patterns: high prior knowledge (HPK) students often made rapid but sometimes error-prone selections, potentially reflecting the expertise reversal effect^58^, whereas low prior knowledge (LPK) students engaged more deliberately, which was associated with fewer errors. These distinct patterns underscore the importance of tailoring scaffolds to learners’ cognitive readiness and demonstrate how temporal analyses can reveal differences that aggregate measures might obscure^6,57^.

Second, our findings contribute to the emerging conceptualization of strategic disengagement as adaptive self-regulation rather than mere disengagement from learning. Traditional perspectives often interpret disengagement negatively, equating it with lack of effort or motivation. However, echoing O’Brien et al.^59^’s agency-driven model, we found that disengagement can represent purposeful, goal-oriented decision-making processes. Specifically, LPK students deliberately disengaged from advanced tasks to manage cognitive load and maintain motivation, while HPK students strategically skipped foundational tasks to optimize efficiency and resource allocation. This aligns with recent theoretical arguments suggesting that strategic pauses and task avoidance can foster metacognitive reflection and better goal alignment, ultimately enhancing learning outcomes^20,59^. Our research operationalizes this reframed understanding of disengagement within adaptive learning contexts, demonstrating how intentional disengagement behaviors can and should be scaffolded productively to support learner agency and sustained engagement over time^17,36^.

Our findings yield several actionable recommendations for the design and deployment of adaptive learning systems and motivational scaffolds within authentic educational settings.

First, adaptive systems should incorporate discrepancy prompts tailored specifically to learners’ prior knowledge (PK) levels. For learners with low prior knowledge (LPK), prompts should encourage reflective engagement with foundational tasks, guiding them to recognize gaps and gradually build confidence and skills^5,7^. For high prior knowledge (HPK) learners, prompts should challenge their intuitive reasoning and heuristics, even after successful task completion or correct answers. For example, the system might ask, “Your solution is correct, but can you simplify or explain the steps you took?” Such discrepancy prompts could stimulate deeper reflection and metacognitive awareness, helping HPK learners refine their understanding and avoid superficial or error-prone strategies^41,51^.

Second, adaptive learning environments should recognize and scaffold strategic disengagement as an intentional and potentially productive choice. Rather than penalizing learners for disengagement, systems could prompt learners to articulate the reasoning behind their decisions to skip or postpone tasks, thereby making disengagement a reflective, metacognitive act. This aligns closely with O’Brien et al.^59^’s reframing of disengagement as agency-driven, highlighting the importance of learners’ active control in managing cognitive load and motivational states. To achieve this, one way is to explicitly implement reflective prompts such as, “Can you explain why you chose to skip this task?” to enhance learners’ metacognitive skills and empower them to regulate their learning paths more strategically. Another way is to incorporate concretized interface elements that encourage learners to articulate reasons behind task disengagement. For example, designing buttons with dropdown selection of brief sentences such as “Skip because I already know the solution”, “Skip because I feel cognitively overloaded” or “Skip because I am confident of my ability to do well” instead of using generic navigation buttons labeled “Next” or “Continue”. Table 6 presents concrete examples of such redesigns.

**Table 6:** Example redesign of prompts and interface elements

Context	Current system	Redesigned prompt/interface element	Intended effect
LPK: Foundational task engagement	“Would you like to review a worked example?”	“Reviewing this worked example can help you spot steps you might miss. Would you like to try it now?”	Encourage reflective engagement and confidence building
HPK: Post-success reflection	No prompt after correct answer	“Your solution is correct, could you explain your reasoning or find a more efficient method?”	Challenge heuristic reliance and deepen reasoning
Strategic disengagement	“Next” or “Continue” button	Add a “Skip task” button with dropdown options: “Already mastered,” “Too easy,” “Feeling overloaded,” “Want to revisit later”	Make disengagement a metacognitive choice; gather actionable learner data
Self-assessment after task	No follow-up	“On a scale of 1–5, how confident are you in applying this method to a new problem?”	Support calibration of understanding to task complexity

Third, our approach underscores the importance of shifting the analytical and practical focus away from simplistic categorizations (e.g., high vs. low performers) toward more nuanced assessments based on learners’ prior knowledge^6,7^. Recognizing that LPK learners might achieve high performance by cautiously building foundational understanding, whereas HPK learners might initially exhibit strong performance yet still benefit from scaffolding that encourages critical self-assessment and deeper reasoning. Therefore, adaptive systems should regularly prompt learners to self-assess and calibrate their understanding against task complexity and personal learning goals, supporting continuous development of strategic choice-making skills.

Fourth, methodologically, we recommend researchers adopt fine-grained analytical techniques, such as process mining, to capture learners’ dynamic choice-making patterns. Utilizing process mining techniques can reveal subtle but significant differences in how students interact with adaptive scaffolds over time, capturing the dynamic evolution of SRL behaviors that traditional analytical methods might overlook^36,57^. Such fine-grained analyses provide robust evidence for designing more precisely targeted and context-sensitive scaffolding interventions.

Finally, our findings have implications for deploying adaptive scaffolds in authentic classroom contexts rather than controlled laboratory settings. While prominent SRL studies such as those involving MetaTutor have largely involved undergraduate students learning complex scientific content in lab environments^15,20^, our study involves adaptive scaffolding within classrooms. This real-world educational setting provides evidence that adaptive motivational scaffolds delivered by pedagogical agents can be feasibly and effectively integrated into everyday learning environments. It also emphasizes the importance of contextually appropriate design considerations, including scalability, practicality, and sustainability of adaptive supports in classroom settings.

We acknowledge several limitations of this study that should be addressed in future work. First, the study was conducted with a relatively small and culturally homogeneous sample of German students in a single, short-duration session (less than one class period). This limits the generalizability of the findings to other cultural or educational contexts. Cross-cultural replications with more diverse learner populations would help determine whether the observed patterns, particularly the interplay between prior knowledge and choice-making, hold across different educational systems and cultural norms.

Second, the current study focused on a single STEM domain (algebra) and a single type of motivational scaffolding, namely prompts framed within Expectancy-Value Theory. Future research could examine other aspects of motivational prompts informed by alternative theoretical frameworks, such as Self-Determination Theory^25^, which emphasizes autonomy, competence, and relatedness, or Goal Orientation Theory^60^, which focuses on mastery and performance goals. Investigating the design and implementation of prompts derived from these theories could provide a richer understanding of how different motivational strategies impact students’ SRL and choice-making behaviors across a broader range of content areas.

Third, the short intervention duration may have constrained the measurable impact of PAs on learning outcomes, as prior studies suggest that motivational effects often emerge over extended or repeated exposure^61,62^. Longitudinal designs could capture how choice-making and strategic disengagement evolve over time and whether sustained PA-supported scaffolding leads to more durable SRL gains.

Finally, the present study measured choice-making through system-logged task engagement decisions. Future work could complement this with multimodal data collection methods, such as eye-tracking, facial expression analysis, or think-aloud protocols, to gain further insights into the cognitive and affective processes driving engagement or disengagement. This richer data could inform more adaptive, context-sensitive scaffolds that respond in real time to indicators of learner state, motivation, and cognitive load.

Overall, our study advances understanding of motivational pedagogical agents within adaptive learning systems by highlighting their nuanced impact on students’ strategic choice-making and conceptual understanding. We demonstrated the critical importance of aligning motivational scaffolds with learners’ prior knowledge. Specifically, we found that low prior knowledge students benefit most from foundational scaffolds, such as worked examples, to build initial confidence, while high prior knowledge students require targeted discrepancy prompts designed to encourage deeper reflection and disrupt heuristic thinking. Our findings also challenge traditional perspectives on disengagement by reframing it as an intentional, adaptive component of self-regulated learning. Practically, our work suggests that adaptive systems should support strategic disengagement, potentially through concretized interface elements that encourage learners to articulate their reasons for skipping tasks. Such design considerations respect learner agency, facilitate reflection, and enhance metacognitive regulation. Moreover, using process mining methods to capture the temporal dynamics of students’ choice-making behaviors allows for more precise and context-sensitive identification of learners’ needs, informing more targeted and timely adaptive interventions. Overall, our research contributes to the ongoing discourse on designing effective adaptive learning environments, offering actionable insights aimed at narrowing achievement gaps among learners with differing prior knowledge levels. Future work should continue exploring personalized scaffolding mechanisms, extending our findings across diverse STEM domains, and leveraging multimodal analytical methods to ensure adaptive technologies effectively support all learners in becoming high performers and executing strategic choice-making behaviors, regardless of their starting point.

## Methods

### The adaptive learning system

The study examines AlgeSPACE, an adaptive learning system designed for early algebra, co-developed with a school teacher over 13 months to support students’ “learning-by-doing” through stepwise problem-solving^63^. AlgeSPACE includes four independent modules focused on solving linear systems of equations, emphasizing distinct strategies such as substitution and elimination methods through real-world examples as well as flexible problem solving using the most suitable strategy. Students can engage with the modules in any order, as they operate independently without predefined sequences.

This study focuses on a single module integrating pedagogical agents (PAs) to scaffold strategic choice-making while fostering conceptual understanding of algebra. Although not a fully developed ITS, the module incorporates adaptive scaffolding features similar to those commonly found in ITS environments. It contains 28 exercises, divided into three types designed to elicit distinct decision-making scenarios and accommodate learners with varying levels of prior knowledge.

*Suitability exercises* allow students to freely select a solution method, after which they can compare their approach to a less efficient hypothetical peer method or re-solve the problem using a system-suggested optimized method. These tasks have moderate cognitive demands and are particularly useful for learners with low prior knowledge (LPK) to build foundational procedural skills while gaining exposure to more efficient strategies through comparison. For high prior knowledge (HPK) learners, these tasks provide opportunities to confirm strategy choice accuracy.

*Efficiency exercises* require students to identify the most efficient method upfront (i.e., requiring the smallest number of solution steps) and then self-explain their rationale. These tasks place higher cognitive demands on learners and are particularly suited to HPK learners who can strategically evaluate methods and articulate reasoning; however, the self-explanation component also supports LPK learners by scaffolding metacognitive awareness even if their initial judgments are less accurate.

*Matching exercises* task students with pairing equations to optimal solving strategies and justifying their choices, emphasizing conceptual understanding of structural features in equations and supporting transfer. For LPK learners, this exercise type offers guided exposure to structural analysis without the full cognitive load of solving, while for HPK learners it reinforces strategic flexibility across problem types.

**Fig. 3:**
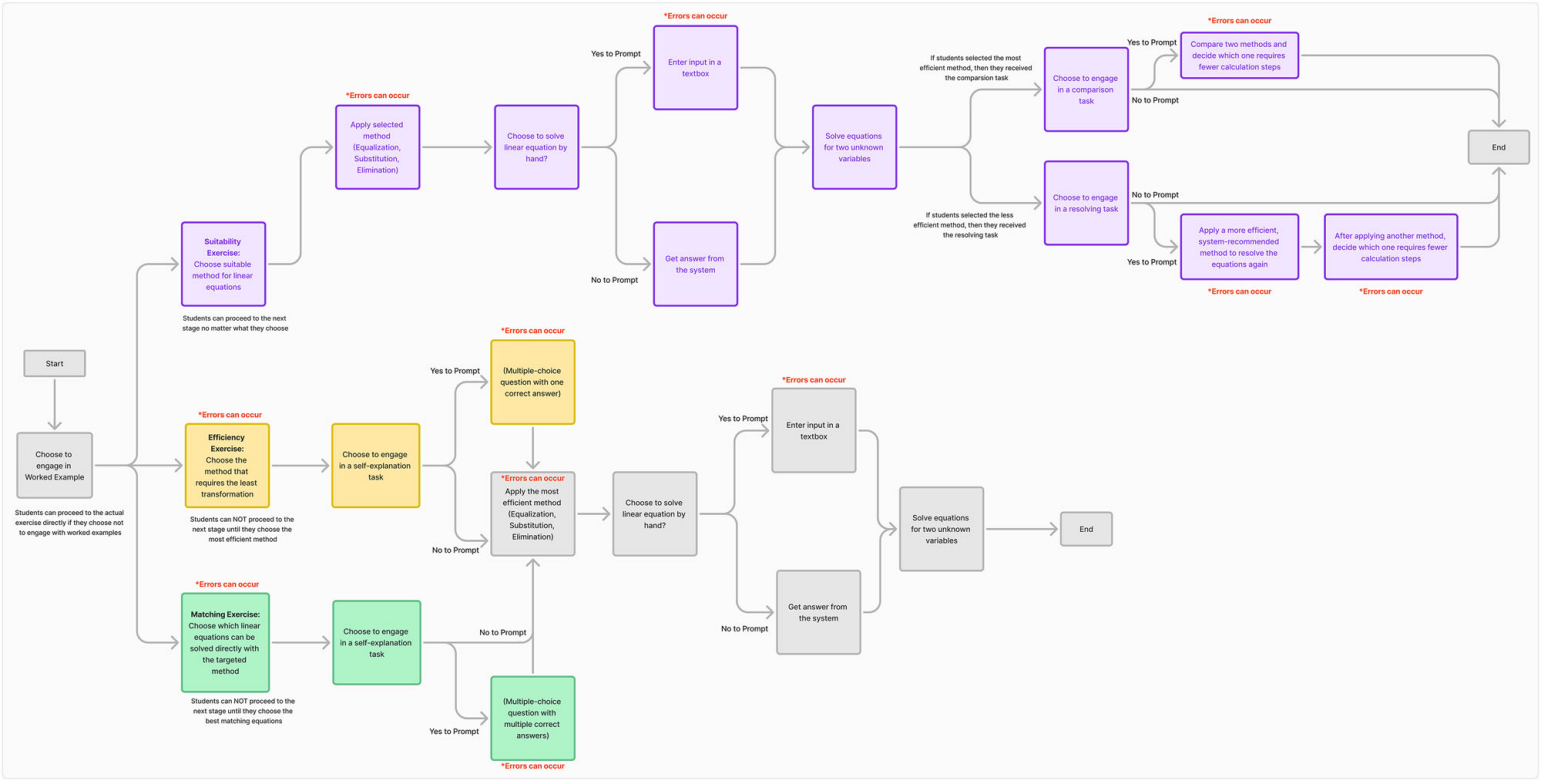
Overview of choice-making workflow. This figure presents the workflow of task choice-making in the AlgeSPACE environment. The diagram shows the sequence of decision points available to students, beginning with the option to engage with a worked example exercise. From this starting point, students proceed into one of several task types represented by colored boxes, with arrows indicating transitions between options. The purple boxes represent suitability exercises, in which students choose to engage in solution-comparison tasks by evaluating and contrasting the suitability of different solutions. The yellow boxes represent efficiency exercises, where students choose to complete a self-explanation task that involves selecting the single most efficient explanation from multiple-choice options. The green boxes represent matching exercises, which also offer opportunities to engage in self-explanation tasks but require students to select multiple correct answers. Each of these exercise types include opportunities to engage in manual calculation tasks, represented as subsequent steps in the workflow. Gray boxes denote common or confirmation steps, such as applying the selected methods to solve equations or ending the session. At each decision point, students may freely decide whether to engage in optional tasks, illustrating the self-regulated nature of the environment.

**Fig. 4:**
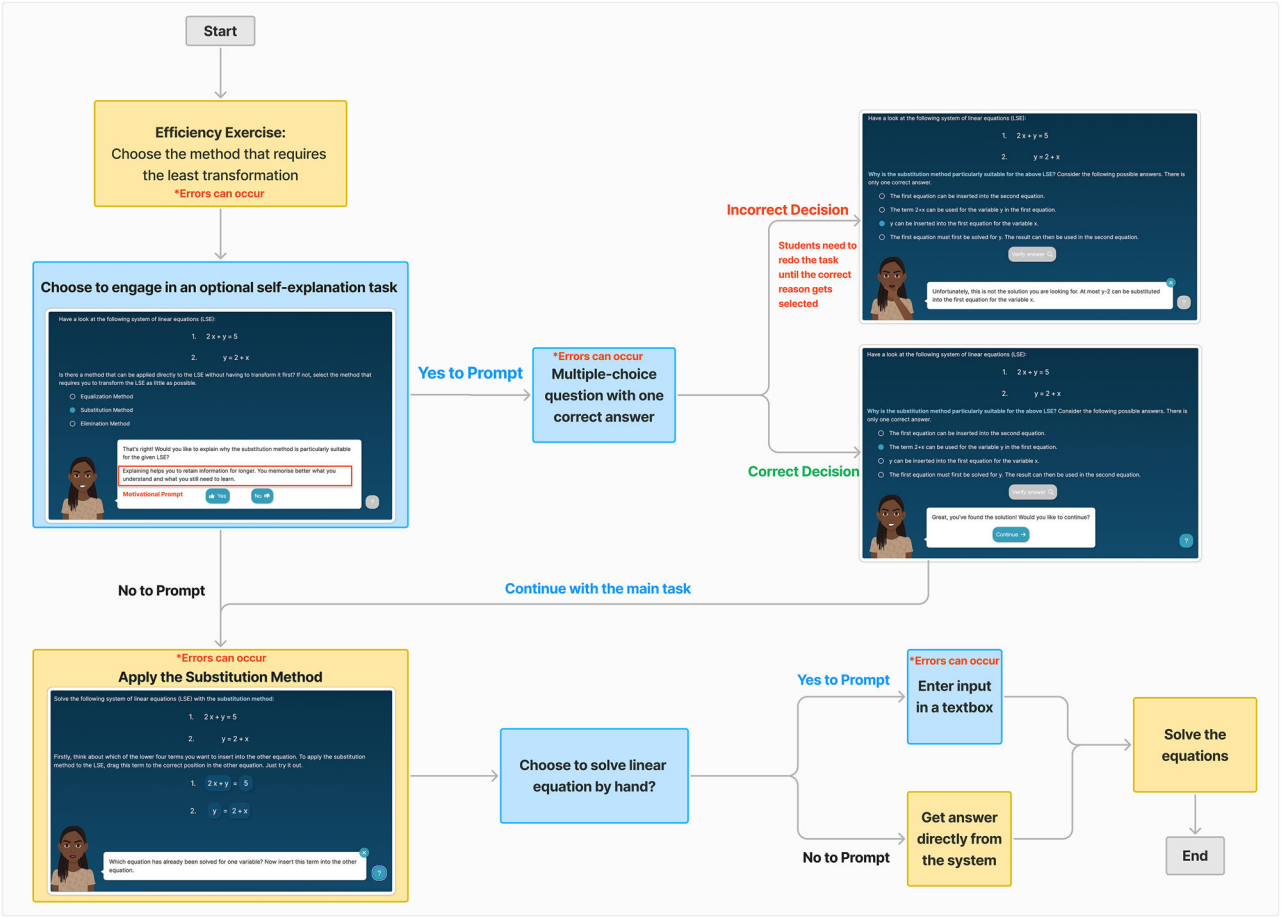
Example interface of an efficiency exercise. This figure illustrates the interface and workflow of an efficiency exercise. At the start, students are prompted to choose the method that requires the least transformation (yellow box, required step). They may then encounter an optional self-explanation activity (blue box, choice-making opportunity), which is presented as a multiple-choice question with one correct answer. Errors can occur at this stage if students select an incorrect explanation option. In such cases, the interface provides immediate feedback (red text, top-right panel), and the student is guided to make another selection. Correct responses (green text, bottom-right panel) allow students to proceed smoothly. In the main task flow, students apply the substitution method (yellow box, required step) to solve the equation. At this point, they face another choice-making opportunity: either attempt the problem manually or receive support from the system. If students opt to solve by hand, they can enter their answer into a textbox (blue box). Errors may occur if an incorrect solution is entered, in which case the system provides corrective feedback before moving to the final solution stage. Alternatively, students may choose to bypass manual input and request the correct answer directly from the system. In all cases, the exercise concludes with the solved equation. Colors are used consistently to encode task function: blue boxes represent choice-making opportunities, yellow boxes represent required or predetermined steps, and gray boxes represent task transitions.

Each exercise presents opportunities to engage with optional cognitive or metacognitive tasks, such as self-explanation, strategy comparison, or manual calculation (see Fig. 3 for an overview of the choice-making workflow). These tasks draw on evidence-based practices for enhancing mathematical learning, such as the benefits of self-explanation for conceptual understanding^64^. For example (see Fig. 4), when solving a system like 2*x* + y = 5 and y = 2 + *x*, students correctly choose the substitution method as the most efficient method and then proceed to the first optional task of self-explanation. Students autonomously choose whether to engage with this self-explanation task by selecting “Yes” or “No,” without penalties or rewards tied to their decisions. There were no evaluative messages indicating whether a choice was “good” or “bad.” Selecting “Yes” led them to complete the prompted self-explanation task, while selecting “No” allowed them to proceed without engaging in self-explanation. This design prioritizes learner autonomy over compliance, allowing progression regardless of choices^3^. The German-language version of the tool was used in this study.

### Participants and experimental conditions

**Fig. 5:**
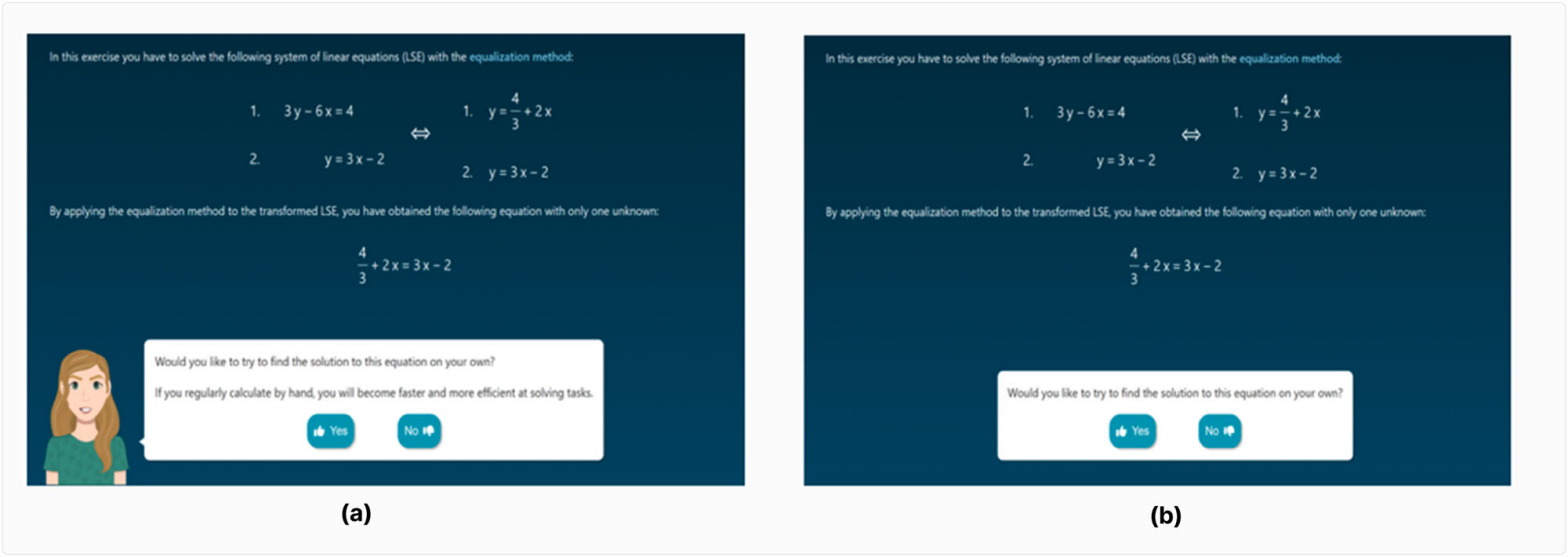
Two versions of the system used in the study. This figure presents the two experimental conditions implemented in the AlgeSPACE environment. Panel **a** shows the agent condition, where a pedagogical agent is displayed in the bottom-left corner of the interface and delivers motivational prompts to encourage engagement with the task. In this example, the agent prompts the student to attempt solving the system of equations by hand and reminds them of the benefits of practicing before receiving automated feedback. Panel **b** shows the non-agent condition, in which the interface is identical except that the agent and its prompts are absent. Students in this condition complete the same tasks without additional motivational scaffolding. Both versions present the same algebraic problems and require students to solve a system of equations using the equation method. The only difference between the conditions is the presence or absence of the pedagogical agent’s visual and motivational prompts.

The study involved 54 students (40 ninth-graders, 14 tenth-graders; typically 14–16 years old) from three classes across two German schools, selected in collaboration with teachers based on curricular relevance. Participants were randomly assigned to one of two experimental conditions in a between-subjects pretest-posttest design (see Fig. 5).

In the Agent Condition, optional task prompts were delivered by an on-screen pedagogical agent that appeared as a human-like character, accompanied by a conversational text bubble containing motivational messages tailored to the specific choice-making scenario (Fig. 5a). The PA’s visual embodiment aimed to create social presence and engagement^50^, and the prompts were phrased in a friendly, encouraging tone to foster rapport and increase learners’ willingness to engage, as predicted by Social Agency Theory^65^. For example, students solving problems manually were reminded that “If you regularly calculate by hand, you will become faster and more efficient at solving tasks.” (Fig. 5a). As shown in Table 7, these prompts are aligned with different choice-making scenarios, such as worked examples (S1_WorkEx), self-explanations (S2_Self-Expl), and comparisons (S3_Comp).

**Table 7:** Pedagogical agents’ motivational prompts for different choice-making scenarios

Scenarios	Definition	Motivational prompts
S1_WorkEx	Engagement with pre-solved worked examples.	“This helps you to remember the topics you have learnt and to solve upcoming tasks more efficiently. ”
S2_Self-Expl	Explaining their own solution strategy (by selecting the most appropriate explanation statement from multiple options).	“When explaining, you think about the best solutions, which strengthens your problem- solving skills”
S3_Comp	Comparing solutions with peers.	“Comparing different methods deepens your understanding of mathematical concepts and how they are related.”
S4_Resolve	Resolving problems using new strategies.	“Trying out different methods shows you that there are often several ways to solve a problem.”
S5 & S6 1^st^ & 2^nd^ Sol	Manually solving for first & second unknown variables	“Manual calculations are required in many exams. Regular practice prepares you for this.”
S7 & S8 Adv_Equ &Adv_Sub	Engagement with complex Equalization or Substitution strategy.	“In this task, you will learn an additional strategy that can also help you with other tasks.”

*WorkEx* worked examples, *Self-Expl* self-explanations, *Comp* comparisons, *Resolve* resolving, *1st & 2nd Sol* 1st solution and 2nd solution, *Adv_Equ* advanced equalization, *Adv_Sub* advanced substitution.

In the Non-Agent Condition, the same system interface was presented without the PA’s visual embodiment or its motivational messaging (Fig. 5b). Students in this condition encountered equivalent task prompts (e.g., “Would you like to compare your solution with a peer’s?”) in concise, instructional text form without additional motivational framing. The absence of PA embodiment and socially framed encouragement removed the interpersonal cues shown to influence learner motivation, perceived support, and persistence^55,66^.

Thus, both conditions featured identical exercises, feedback, and choice-making opportunities. The key difference lay in how optional task prompts were delivered: in the Agent Condition, prompts were accompanied by a human-like pedagogical agent and framed with socially engaging, motivational language; in the Non-Agent Condition, prompts were presented as concise, instructional text without agent embodiment or motivational framing.

### Measures

*Pre-Post Tests.* Students’ conceptual understanding of linear systems was assessed through counterbalanced pre- and posttests, each containing three exercises measuring three constructs, including method selection accuracy (identifying optimal solving strategies such as substitution, equalization, or elimination^67^), mathematical justification (explaining rationale for method choice^68^), and procedural fluency (solving system correctly^69^). Each exercise followed a three-part format (method selection, justification, and solution), scored as 1, 2, and 3 points respectively (max 18 points per test). For more details of the test, see Supplementary Information (Supplementary Note 1).

*Prior Knowledge.* Pretest scores (median = 6.75, M = 7.39) were used to classify students into high- (*n* = 24) or low-prior-knowledge (*n* = 25) groups via median split. A t test confirmed significant differences between groups (t(47) = −2.13, *p* < 0.05).

*Error Rates*. Errors (e.g., incorrect method selection, incorrect choice of reasons for the self-explanation tasks) were logged to assess conceptual misunderstandings. Error rates were calculated as total errors divided by exercises attempted. Errors can occur under different choice-making scenarios (see Fig.3. red marks “Errors can occur”).

*Choice-Making Behaviors*. Engagement with optional tasks (e.g., self-explanation, strategy comparison) was measured by choice-making frequency - the proportion of “Yes” responses to total choice-making opportunities (range: 0–1).

### Procedure

The study was conducted during regular school hours in a university classroom for approximately an hour with a total of 54 participating students. All participants were secondary school students under the age of 18. Prior to the study, written informed consent was obtained from their legal guardians, and student assent was collected on the day of participation. Ethical approval was granted by the authors’ institutional review board (No. 23-12-09) before data collection began.

During the study, participants began with a 10-min pretest assessing their baseline understanding of linear equations. This was followed by a 5-min interactive tutorial introducing the system. Students then engaged in a 25-min learning session using their own, school-provided iPads, working independently in their assigned condition. Afterwards, they completed a 10-min posttest with isomorphic problems from pretest to measure learning gains and a brief questionnaire on their perceptions of the tool. Interaction data which includes choice responses (Yes/No), answer correctness, solution steps, and time spent were logged automatically by the system.

Of the 54 students who participated, five students were excluded from the analyses due to incomplete posttests or disengagement (e.g., skipping all optional tasks), resulting in a final analytic sample of 49 students (Agent Condition: *n* = 24; Non-Agent Condition: *n* = 25).

### Data analysis

To address RQ1 (impact of pedagogical agents on conceptual understanding) and RQ2 (influence of PAs on choice-making behaviors), we conducted ANCOVAs (to compare posttest scores and error rates between conditions, controlling for their prior knowledge, measured through pretest scores). We also conducted two-way ANOVAs to examine the interaction effect between condition (Agent/Non-Agent) and prior knowledge (High/Low) on posttest scores, learning gains, and error rates. Post-hoc pairwise comparisons (Tukey-adjusted) clarified significant differences between subgroups (e.g., Agent High vs. Non-Agent Low on posttest scores). For RQ3 (dynamic choice-making processes over time), Lag Sequential Analysis (LSA) identified temporal action patterns using z-scores and Yule’s Q values, while Process Mining visualized macro-level regulatory trajectories across prior knowledge groups.

The system logged detailed interaction data throughout the learning process, including students’ engagement with optional tasks (e.g., “No to worked examples”, “Yes to self-explanation”), solution strategy selection (e.g., “selected Equalization”, “selected Substitution”), problem-solving steps (e.g., “DRAG TERM from SECOND RIGHT and DROP for VAR y, SUCCESS”), error incidents (e.g., “selected option 3, FAILURE”), and success outcomes of their problem solving (e.g., “SUCCESS”, “FAILED” flags).

To translate these raw interaction logs into meaningful learning decision actions, we developed a systematic mapping scheme tailored to the task contexts. This approach follows the log-to-action mapping procedures in previous learning analytics studies^34,57,70^. In our study, each logged event was interpreted based on a combination of recorded fields and external task-specific references. The mapping was implemented as a deterministic, rule-based process in which each event type was assigned to a predefined category according to fixed criteria derived from system parameters (e.g., success/failure flags, task IDs) and task documentation. Because this process applies consistent rules to structured log data without subjective coder judgement, inter-coder reliability measures (e.g., Cohen’s κ) are not applicable. To ensure accuracy, the mapping rules were iteratively tested on a sample of events, cross-checked against task specifications, and refined until all cases were correctly assigned. Actions involving voluntary engagement (e.g., whether to engage in optional self-explanations or worked examples) were coded as explicit “choices” (Yes/No decisions: YesToExtra, NoToExtra, YesToPrompt, NoToPrompt). In contrast, method selections within required problem-solving tasks were categorized separately as either “AttempSuccess” or “FailedAttempt,” depending on correctness relative to task documentation and associated error logs. Through several iterations, we mapped the raw log event types to eight learning decision actions (see Table 8).

**Table 8:** Mapping scheme for learning decision actions

Actions code	Description	Column	Map rules
NoToExtra	Not engage with worked examples or advanced exercise	Worked Examples Choice, Tip Choice	Contain str ‘No’
YesToExtra	Engage with worked examples or advanced exercise	Worked Examples Choice, Tip Choice	Contain str ‘Yes’
NoToPrompt	Not engage with prompt request	First Solution Choice, Second Solution Choice, Comparison Choice, Self Explanation Choice, Resolving Choice	Contain str ‘No’
YesToPrompt	Engage with prompt request	First Solution Choice, Second Solution Choice, Comparison Choice, Self Explanation Choice, Resolving Choice	Contain str ‘Yes’
FailedAttempt	Incorrect choice of methods or transformation actions steps	First Solution Actions, Second Solution Actions, Self Explanation Actions	Does not contain ‘RESOLVE’, Action Errors column = 1
		Suitability Transformation Actions	Contain ‘RESOLVE’, Action Errors column = 1
		Efficiency Selection Actions, Elimination Actions, Equalization Actions, Substitution Actions, Suitability Transformation Actions, System Matching Actions, Transformation Actions,	Does not contain ‘RESOLVE’, Action Errors = 1
AttemptSuccess	Correct choice of methods or transformation actions steps	First Solution Actions, Second Solution Actions, Self Explanation Actions	Action Errors column = 0
		Suitability Transformation Actions	Contain ‘RESOLVE’, Action Errors column = 0
		Efficiency Selection Actions, Elimination Actions, Equalization Actions, Equation Selection, Substitution Actions, Suitability Transformation Actions, System Matching Actions, Transformation Actions	Does not contain ‘RESOLVE’, Action Errors = 0
		Elimination Actions, Equalization Actions, Substitution Actions	Contain ‘RESOLVE'
IncorrectDecision	Incorrect choice of prompt’s questions	Comparison Decision	If Decision = Incorrect, Action Encoded contain ‘COMPARISON’
		Resolve Conclusion Decision	If Decision = Incorrect, Action Encoded contain ‘INITIAL'
CorrectDecision	Correct choice of prompt’s questions	Comparison Decision	If Decision = Correct, Action Encoded contain ‘INITIAL'
		Resolve Conclusion Decision	If Decision = Correct, Action Encoded contain ‘RESOLVE'

Tip Choice = advanced exercise.

In total, the analysis resulted in 5144 labeled decision actions, organized into 49 sequences, each representing a complete interaction sequence of a single student across all tasks within the adaptive system.

To analyze differences in interaction sequences between high- and low-prior-knowledge learners, Lag Sequential Analysis (LSA) was applied to the coded decision-action data. This method estimates the likelihood of consecutive actions by analyzing overlapping samples, evaluating the probability of one event following another^71^. The purpose of LSA in this study was to identify sequences of student behaviors that occur with greater frequency than would be expected by chance, thereby uncovering significant patterns in task engagement and decision-making processes.

The term ‘lag’ in LSA denotes the temporal relationship between events, with ‘lag 1’ indicating direct transitions where one event immediately follows another, and ‘lag 2’ representing indirect transitions with an intervening action, suggesting more complex or delayed strategies or decision-making process. Sequences are deemed statistically significant if they achieve z-scores ≥ 1.96 (*p* = 0.05). However, as z-scores alone may not confirm the robustness of a pattern, Yule’s Q was also calculated^72^. Yule’s Q, a transformation of the odds ratio scaled between −1 and +1, measures the strength of association. Significant z-scores paired with Yule’s Q values ≥ 0.30 indicate meaningful relationships.

Significant sequences at lag 1 were identified first, with three-event chains considered significant only if both the lag 1 and lag 2 transitions demonstrated statistical significance^73^. Due to the large number of coded actions and the increased likelihood of Type I errors in significance testing, Pearson chi-square (χ²) tests were performed to validate the overall significance of observed transitions^71^. A significant χ² result justified further analysis of individual sequences, using adjusted residuals and z-scores to assess their validity.

The *LagSequential* R package^74^ was used for this analysis, computing transition probabilities, chi-square values, z-scores, Yule’s Q, and the count of code transitions. This approach revealed how high- and low-prior-knowledge learners differed in their engagement and regulatory processes, particularly in response to system prompts.

Process mining was used to model learners’ interaction pathways, capturing how underlying cognitive and regulatory strategies shape sequential behaviors^56^. The analysis was performed using Fluxicon Disco (https://fluxicon.com/disco/), a widely used tool for process mining in education^36,75^. Results were visualized through Heuristic Net graphs, highlighting event frequencies and dependencies, providing insights into the sequential structures and regulatory strategies that characterize student interactions across different prior knowledge groups. This approach provided macro-level insights into how prior knowledge moderates the impact of system scaffolding on self-regulated learning.

## Supplementary information

Supplementary Information

## Data Availability

Due to privacy restrictions, the raw student log data cannot be shared publicly. Derived data supporting the findings of this study are available from the corresponding author upon request.

## Code availability

The analysis scripts are available from the corresponding author upon request.
