# Supplementary material for: Choice-making in an adaptive learning system with motivational pedagogical agents
Source: NPJ Sci Learn. 2025 Nov 18;10:77. doi: 10.1038/s41539-025-00366-7 (PMC12627807; doi:10.1038/s41539-025-00366-7)
Supplement: Supplementary file 1 — Supplementary Information [file 41539_2025_366_MOESM1_ESM.docx]

**Supplementary Information**

**Supplementary Table 1. Motivational Prompts Categorized by Expectancy-Value Theory (EVT) Constructs**

| **Prompts** | **EVT Construct** |
| --- | --- |
| **Prompts for Solving Linear Equations by Hand** | |
| If you calculate by hand, you understand the individual steps better and can recognize and correct errors more quickly. | Utility value |
| By calculating by hand, you will better understand how mathematical concepts and rules are related. | Intrinsic value |
| Manual calculations are required in many exams. Regular practice prepares you optimally for this. | Attainment value |
| If you can solve simple equations by hand, you will be better prepared to tackle complex problems. | Expectancy for success |
| Solving equations by hand makes you less dependent on technology. | Utility value |
| **Prompts for Comparing Strategies** | |
| The comparison shows you how different approaches can lead to the same result. This broadens your understanding and shows you new perspectives. | Intrinsic value |
| Each method has its advantages and disadvantages. By comparing them, you will learn which method is best used when. | Utility value |
| Comparing different methods deepens your understanding of mathematical concepts and how they are related. | Intrinsic value |
| In exams, you often need to know different approaches. Comparing different methods prepares you optimally for this. | Attainment value |
| If you know and can compare several methods, you are more flexible and can choose the best method depending on the problem. | Utility value, expectancy for success |
| **Prompts for Applying Another Strategy** | |
| Trying out different methods shows you that there are often several ways to solve a problem. | Intrinsic value |
| An alternative method can be faster and more efficient. By practicing different approaches, you will find the method that suits you best. | Utility value |
| By trying out a different method, you discover new ways to solve the problem. This will help you to find out which method is quickest or easiest, which will help you to work more efficiently. | Utility value |
| If you master different methods, you can react flexibly to different problems. | Expectancy for success |
| If you have mastered different methods, you will be better prepared for more complex problems. You can then choose the method that best suits the problem at hand. | Expectancy for success |
| If you try out a different method, you may notice mistakes that you previously overlooked. This will help you to work more precisely and improve your skills. | Utility value |
| **Prompts for Self-Explaining** | |
| By explaining solutions, you understand the concepts much better and realize how everything is connected. | Intrinsic value |
| When you explain your solutions, you can immediately see where you still have uncertainties and what you still need to practice. | Utility value |
| Explaining helps you to retain information for longer. You memorize better what you understand and what you still need to learn. | Utility value |
| When explaining, you think about the best solutions, which strengthens your problem-solving skills. | Intrinsic value |
| Explaining helps you to recognize connections between different topics and develop a deeper understanding. | Intrinsic value |
| **Prompts for Other Voluntary Exercises** | |
| In this task, you will learn an additional strategy that can also help you with other tasks. | Utility value |
| In this task, you will learn how to use the substitution method even more efficiently. This will also prepare you well for more difficult tasks. | Utility value |
| **Prompt for Reviewing Worked Examples** | |
| This helps you to remember the topics you have learnt and to solve upcoming tasks more efficiently. | Utility value |

**Supplementary Note 1. Pre-posttest**

Please read the following information carefully:
• This activity contains **3 short exercises** on the topic of solving systems of linear equations.
• You have **10 minutes** in total to complete the exercises. Try to answer as many problems as possible. If you encounter any difficulties with an exercise, proceed to start another one and possibly come back later to this exercise. We will remind you of the remaining time.
• Your performance in these exercises will not affect your grade at school in any way, making mistakes is only normal and expected. Moreover, all activities are anonymized, i.e. we cannot trace the answers back to you.
• Feel free to take notes next to the exercises. Wait for us to signal you to start.

**Exercise 1:** Have a look at the following system of linear equations:

*y = x −* 1

*y =* 2*x −* 4

(a) In your opinion, which of the following three methods is best suited to solve this system?

Select one answer:
⃝ Equalization method
⃝ Substitution method
⃝ Addition method

(b) Please explain your answer from part (a):

(c) Solve the system with a method from part (a) of your choice. Remember, to write down the solution point.

**Exercise 2:** Have a look at the following system of linear equations:

3*x* + 4*y* = − 2

− 3*x* − 5*y* = 1

(a) In your opinion, which of the following three methods is best suited to solve this system?

Select one answer:
⃝ Equalization method
⃝ Substitution method
⃝ Addition method

(b) Please explain your answer from part (a):

(c) Solve the system with a method from part (a) of your choice. Remember, to write down the solution point.

**Exercise 3:** Have a look at the following system of linear equations:

*x* - 5*y* = 4

*y* = 2*x* + 1

(a) In your opinion, which of the following three methods is best suited to solve this system?

Select one answer:
⃝ Equalization method
⃝ Substitution method
⃝ Addition method

(b) Please explain your answer from part (a):

(c) Solve the system with a method from part (a) of your choice. Remember, to write down the solution point.
